# Is it Better to Intermarry? Immigration Background of Married Couples and Suicide Risk Among Native-Born and Migrant Persons in Sweden

**DOI:** 10.1007/s10680-023-09650-x

**Published:** 2023-03-08

**Authors:** Anna Oksuzyan, Sven Drefahl, Jennifer Caputo, Siddartha Aradhya

**Affiliations:** 1 Chair of Demography and Health, School of Public Health, Bielefeld University, 33615 Bielefeld, Germany; 2 Max Planck Institute for Demographic Research, Rostock, Germany; 3 Demography Unit, Institute of Sociology, Stockholm University, Stockholm, Sweden; 4 Center for Health and the Social Sciences and Department of Sociology, University of Chicago, Chicago, USA; 5 Westat, Rockville, MD USA

**Keywords:** Suicide, Immigrant, Intermarriage, Mental health register data, Sweden

## Abstract

Marriage is protective against suicide across most populations, including for persons of different ethnicities and immigrant backgrounds. However, the well-being benefits of marriage are contingent upon marital characteristics—such as conflict and quality—that may vary across spousal dyads with different immigration backgrounds. Leveraging Swedish register data, we compare suicide mortality among married persons on the basis of their and their spouse’s immigration backgrounds. We find that relative to those in a native Swede-Swede union, Swedish men married to female immigrants and immigrant women married to native men are at higher risk of death by suicide, while immigrants of both genders who are married to someone from their birth country have a lower risk of suicide mortality. The findings support hypotheses about the strains that may be encountered by those who intermarry, as well as the potential selection of individuals into inter- and intra-ethnic marriages.

**Supplementary Information:**

The online version contains supplementary material available at 10.1007/s10680-023-09650-x.

## Introduction

According to the World Health Organization, approximately 800,000 deaths by suicide occur every year worldwide (WHO, 2018). Suicide was recognized as a social problem by Durkheim in (1897), who noted that social integration, including marriage, appeared to be protective against suicide—a finding that continues to be replicated in more recent research (Stack, 1990; Stack & Wasserman, 1993). Research documents variations in these benefits across social groups, such as by gender (Denney et al., 2009; Luoma & Pearson, 2002), ethnic background (Spallek et al., 2015; Wadsworth & Kubrin, 2007), and across nations (Mayer & Ziaian, 2002; Zhang, 2010). However, variation in suicide risk by the immigration backgrounds of spousal dyads––that is, marriages involving individuals from different or the same ethnic background (hereafter, intermarriages and intramarriages, respectively) is understudied. Intermarrying with native-born populations is considered to be one of the strongest indicators of immigrants’ integration into a host country (Dribe & Lundh, 2008; Furtado & Song, 2015; Meng & Meurs, 2009). At the same time, investigators find that intermarriages are less stable than intramarriages owing in part to strains that include familial cultural conflict, experiences with discrimination, and reduced marital quality (Dribe & Lundh, 2012; Kalmijn et al., 2005). Despite increased interest in the social and health implications of inter- and intra-marriages, studies exploring whether it is more beneficial for mental health to marry someone of the same or a different ethnicity are scarce (Eibich & Liu, 2021; Milewski & Gawron, 2019).

Leveraging Swedish registry data, we investigate differences in suicide risk across inter- and intramarried individuals of both Swedish and immigrant origins. With the first research question we ask *does the risk of suicide among married men and women of both Swedish and immigrant origins depend on whether they are married to an immigrant or a native-born person?* Given that studies show that ethnic intermarriages may be characterized by cultural dissimilarities, marital discord, and instability, we hypothesize that both partners in intermarriages between persons of Swedish and immigrant origin will have a higher risk of suicide relative to Swedes married to other natives. We also expect that immigrants married to other immigrants from different countries of origin will have greater risk of suicide, although perhaps to a lesser extent, since they share the experience of immigration. Additionally, consistent with research showing that healthier individuals are more likely to immigrate and that spouses with the same ethnic background are less likely to divorce than partners with dissimilar backgrounds, we hypothesize that immigrants married to other immigrants from their own countries will exhibit the lowest suicide risk of all groups.

Our second research question asks *among married immigrants, does the risk of suicide differ for those married to a Swede or an immigrant from a different country compared to those married to another immigrant from the same country?* When examining the immigrant population only, we hypothesize that immigrants married to Swedes will have the highest risk of suicide of all three groups. Because partners have shared experiences of immigration, we expect that cultural conflicts may be less salient in immigrant intermarriages, even if both partners are from different countries of origin, than they are in intermarriages involving a Swedish partner. The analyses focusing on the immigrant population allow us to adjust for confounders pertinent to immigrants only, specifically age at immigration and region of origin. Including a region of origin measure helps to account for important cultural background differences in beliefs about suicide, marriage and gender.

## Background

### Inter- and Intra-Ethnic Marriages and Well-Being

Durkheim’s classic social study on suicide (1897) contended that it is often driven by an extreme lack of integration with society. He argued that marriage is one of the most important indicators of social integration, noting that the frequency of suicide was much lower among the married. Later research provided further compelling evidence that marital bonds are linked to lower suicidal behavior (Kposowa, 2002; Luoma & Pearson, 2002; Martikainen & Valkonen, 1996; Stack, 1990). Substantial benefits of being married—which is associated with healthy behaviors and provides individuals with economic, emotional, and social support (Ross, 1995; Umberson, 1987; Waite, 1995)—have been found for a host of other mental and physical health indicators (Kravdal, 2001; McLaughlin et al., 2011; Simon, 2002). Even so, the quality of a marriage conditions its relationship to well-being. Marriage can introduce interpersonal stressors, such as relationship conflict, that have been linked to poorer health and increased risk of suicide attempts (Kaslow et al., 2000; Robustelli et al., 2015; Umberson et al., 2006).

Prior studies show that the health benefits of marriage vary across individual characteristics, including race/ethnicity (Elwert & Christakis, 2006; Johnson et al., 2000; Martínez et al., 2016) and gender, with research suggesting that men benefit more than women (Denney et al., 2009; Gove, 1973; Kposowa, 2002; Luoma & Pearson, 2002). However, questions about whether it is more beneficial for health to be married to someone from the same or a different culture remain under-studied. The literature supports conflicting expectations.

On the one hand, it may be that intermarriage benefits both native-born and immigrant persons as it increases cultural capital, which may enhance individuals’ sense of meaning and well-being (Rodríguez-García, 2015). Immigrant populations may especially benefit from marrying native spouses. Living with native-born spouses can improve language skills, access to social networks, and knowledge about the culture, healthcare, and social systems of the host country, all of which may improve immigrants’ well-being. Accordingly, intermarriage has been associated with better economic outcomes among immigrants in Sweden (Dribe & Lundh, 2008; Elwert & Tegunimataka, 2016; Tegunimataka, 2017), Denmark (Elwert & Tegunimataka, 2016), France (Meng & Meurs, 2009), and the US (Furtado & Song, 2015). The limited prior research provides some evidence for the health benefits of intermarriage, but findings are modest. A German-based longitudinal study revealed a greater short-term increase in life satisfaction among native-born German women (but not native-born German men) who intermarried as compared to their counterparts who married another German, but the added benefit disappeared in the long-term (Potarca & Bernardi, 2021). A study based on data from nine European countries found better mental health among intermarried than intramarried immigrants, but not their native-born spouses (Milewski & Gawron, 2019).

On the other hand, intermarriage may be accompanied by cultural conflicts between spouses and extended family members, experiences with discrimination, and reduced marital quality, all of which are linked to poorer health. Interethnic marriages are less stable than intramarriages, which scholars have partly attributed to marital discord arising from sociocultural differences in values, norms, and communication styles (Dribe & Lundh, 2012; Kalmijn et al., 2005; Milewski & Kulu, 2014). Indeed, findings suggest that the risk of divorce within interethnic marriages increases with cultural differences between partners (Dribe & Lundh, 2012; Kalmijn et al., 2005). Intermarriages that are characterized by conflict and instability may take a toll on well-being, as suggested by research showing that cultural conflict increased depressive symptoms among both spouses in Turkish-British marital dyads (Baltas & Steptoe, 2000). At least one prior study supports the hypothesis that intermarriage predicts poorer mental health than intramarriage, indicating that both partners in intermarriages between native German women and immigrant men had worse mental health than intramarried native-born persons of their gender (Eibich & Liu, 2021).

Together, these studies suggest that the relationship between intermarriage and mental health differs by gender and nativity. It may also be the case that selection processes, whereby men and women with (un)healthy behaviors are more likely to form interethnic spousal dyads, contribute to health (dis)advantages of intermarried people. Potarca and Bernardi (2021) showed that relative to their intramarried peers, immigrant men married to German women reported high levels of life satisfaction prior to marriage. However, at least one US-based study indicates that the health gap between whites in same-race and interracial marriages remains after accounting for differences in health behaviors (Yu & Zhang, 2017).

### Suicide Behaviors across Nations, Gender, and Immigration Background

Suicide behaviors vary across nations, gender and immigration background in complex ways that are important to consider in an exploration of the links between inter- and intramarriages and suicide mortality. There is substantial cross-national variation in suicide rates. For example, in 2017 suicide rates across countries of the European Union were highest in the Baltic countries, Slovenia, and Hungary. They were also relatively high in Sweden and other Nordic countries, and lowest in Southern European countries, e.g., Greece, Italy and Spain (Andrés, 2005; OECD & European Union, 2020). These patterns likely reflect complex cross-national differences in material resources, as well as cultural and religious beliefs.

Studies also find that men consistently have higher rates of suicide than women across nations and cultural contexts (Andrés, 2005; Pampel, 1998). However, studies examining the relationship between gender inequality and the male-to-female sex ratio of suicide rates have revealed mixed findings. While some cross-sectional findings suggest that there is no relationship between gender equality and the sex ratio of suicide rates (Shah, 2008), other studies have found that it predicted higher male-to-female suicide rate ratios (Chang et al., 2019). Prior research has also found that gender equality was related to higher suicide rates for both men and women (Mayer, 2000). However, a more recent longitudinal study covering 87 countries showed that increasing gender equality was associated with a significant reduction in within-country suicide rates for women (Milner et al., 2020). Although the relationship between the gender equality index and male suicide rates was also negative in this study, it did not reach a statistically significant level. These findings support the hypothesis that having multiple roles reduces social isolation and increases economic resources contributing to the improved psychological well-being (Milner et al., 2020).

Findings about differences in suicidal behavior between immigrants and non-immigrants in Sweden and other Nordic countries similarly reveal patterns that vary by country of origin and gender (Spallek et al., 2015; Wadsworth & Kubrin, 2007). For example, Westman et al. (2006) showed that male Finnish immigrants and female immigrants from Eastern Europe had an elevated suicide risk compared to Swedish men and women, respectively, while immigrants from the Middle East and male immigrants from Southern Europe had lower risks. They speculated that long-standing discrimination against Finns living in Sweden, such as not being allowed to speak their native language, and the greater vulnerability of Eastern European women to broken social ties might underlie these gender-specific patterns by country of origin. In contrast, in Norway, Eastern European immigrant men but not women were at a lower risk of suicide than native-born persons (Puzo et al., 2017), which may reflect a stronger health selection of male immigrants arriving for work and their trailing spouses. Johansson et al. (1997) showed substantial variations in suicide risk among both male and female immigrants to Sweden even within the countries of the same East European region, highlighting the likely importance of gender differences in suicide behaviors among immigrants from some regions. A review by Spallek et al. (2015) notes some generalizable patterns among the varied findings. Specifically, immigrants from Northern and Eastern European countries have higher risks of suicide death, while those from Southern Europe tend to have lower suicide risks compared to host populations. A positive correlation between the suicide attempt rates of immigrants to European countries with the rates in their countries of origin suggests that the cultural and religious characteristics of immigrants’ homelands may either shield against or aggravate suicidal behavior (Lipsicas et al., 2012).

Immigrants’ varying levels of social integration and socioeconomic status may also contribute to differences in suicide rates between them and native-born persons. There is compelling evidence that members of lower socioeconomic status groups have higher risk of suicide than their better-off peers (Li et al., 2011). Although immigrants’ levels of social and economic integration within Swedish society vary across country of origin, on average they have lower socioeconomic standing than native-born persons. Until the 1990s, employment rates among immigrant men were similar to those of Swedes, and even higher among foreign-born than native-born women (Gustafsson, 2020). However, from the 1990s onwards, employment rates among foreign-born men and women declined rapidly. In 2016, unemployment rates were 3% among the native-born population compared with 14–15% among foreign-born persons. Furthermore, a greater proportion of foreign-born women were not in the labor force––neither employed nor actively searching for a job––than native-born women. Studies also show that employed immigrants earn less on average than native-born Swedes, and that immigrants often live in segregated neighborhoods (Gustafsson & Österberg, 2018; Wimark et al., 2019). These disadvantages may lead to poorer mental health, lower human capital, and social tension (Raphael et al., 2020). To account for the better economic outcomes of intermarried immigrants, our analyses adjust for education, income and employment.

### Immigration Patterns in Sweden

Although it is now a multi-ethnic country, Sweden was relatively homogenous until the 1960s, when just 4% of the population was foreign-born (Statistics Sweden, 2021). The majority of foreign-born people living in Sweden in the 1960s were labor immigrants from other Nordic countries, Italy, Greece, Yugoslavia, and Turkey (Swedish Migration Agency, 2021). Non-Nordic labor immigration was restricted in the 1970s, while non-Nordic family reunion immigration and refugee immigration, particularly from Chile, increased. In the 1980s, the number of asylum seekers from the Middle East, the Horn of Africa, and the former Eastern Bloc countries increased, which continued into the 1990s due the Yugoslav Wars. In the 2010s, immigration to Sweden was predominated by refugees from Syria. In 2016, 17.9% of the population was foreign-born, and 56.8% of these residents had Swedish citizenship (Statistics Sweden, 2021).

## Data and Methods

This study utilized register data that contain a wide variety of characteristics for all Swedish residents, including demographic characteristics, socioeconomic status, and cause-of-death. These registers have nationwide coverage with low risk of inaccurate linkages across registers (Ludvigsson et al., 2009). The study population consisted of people aged 18 or older living in Sweden between January 1, 1991 and December 31, 2016. Individuals entered the study from the month during this window that they became married or the month that they immigrated if they were married when they arrived to Sweden. All individuals were followed until death, emigration, or December 31, 2016; whichever came first. The data were interval-censored, meaning individuals could re-enter the study population at re-immigration.

The main variable of interest––*immigration background of married couples* (hereafter “marriage type”)––was defined in the following categories: Swedish-Swedish, immigrant-Swedish, immigrant intermarriage (i.e., immigrant spouses from different countries of birth), immigrant intramarriage (immigrant spouses from the same country of birth), and Swedish-immigrant. The latter group represents the same spousal dyads as immigrant-Swedish marriages, but the mortality hazard is estimated for the Swedish spouse rather than the immigrant spouse.

We focus on intact married couples to avoid the potential effect of marital disruption through divorce or widowhood on suicide mortality (Martikainen & Valkonen, 1996). To control for differences in socio-demographic composition, we adjust the following covariates: education (primary, secondary, post-secondary), income (tertiles of individual disposable income), and employment (employed vs. not working). The latter three variables are treated as annually time-varying. Based on earlier findings that presence of children, particularly young children, in the household is protective against self-destructive behavior (Qin & Mortensen, 2003; Veevers, 1973), we also control for the presence of a minor child in the household. Because experiences surrounding migration and challenges in the host country and their role in shaping immigrants’ health may differ between men and women (Llácer et al., 2007; Malmusi et al., 2010), all analyses were conducted in sex-specific samples.

Our analysis employed Cox proportional hazard models (Cox, 1972), with death due to suicide as the failure event. The proportional hazards assumption for each covariate was tested on the basis of Schoenfeld residuals after fitting a model. We found no major violations in the proportionality assumption for the main variable of the interest or the covariates. The baseline hazard is a function of age. In the first model we include marriage type to assess whether suicide mortality differs across different marriage groups. We add socioeconomic characteristics in Model 2 and the presence of a minor child in Model 3 to assess whether the inclusion of these controls modifies the observed relationships between marriage type and suicide mortality.

In the second step, we restrict analyses to the immigrant population to assess variations in suicide risk among immigrants in different marriage types. The variable *country of birth* was recoded into three larger groups in order to retain a sufficient number of events within each marital composition group. The first group includes immigrants from higher income countries, including Nordic countries (Denmark, Finland, Iceland, Norway), Western European countries (Andorra, Austria, Belgium, Cyprus, France, Germany, Greece, Ireland, Italy, Liechtenstein, Luxembourg, Malta, Monaco, The Netherlands, Portugal, San Marino, Spain, Switzerland, United Kingdom, Vatican City), North America (USA and Canada), Australia, and New Zealand. The second country-of-origin category groups together immigrants from predominantly Eastern European countries (Bosnia and Herzegovina, Bulgaria, Croatia, Czech Republic**,** Estonia, Hungary, Latvia, Lithuania, Macedonia, Montenegro, Poland, Romania, the Russian Federation and other countries of the former Soviet Union, Serbia, Slovakia, Slovenia, and Albania). The third group includes immigrants from non-Western countries (Table 1), predominately Asia and the Middle East. To further explore whether country-of-origin-specific characteristics underlie our results, we performed analyses splitting the immigrant sample between those originating in Western and non-Western countries. We also conducted analyses splitting the immigrant sample into those who arrived in Sweden before and after age 18 to help shed light on whether immigrants’ degree of social integration in Sweden—as indicated by whether they spent some of their formative childhood years in the country (Aslund et al., 2009; Söhn, 2011)—influenced the relationship between their marriage type and suicide behaviors. To gain additional insights on whether selection into marriage type and/or health selection among immigrants play a role in explaining the relationship between marriage type and suicide risk, we performed sensitivity analyses with all-cause mortality as an outcome in the total and immigrant populations. Finally, given previous findings that the protective effect of parental status differs for men and women in that having a young child is protective of suicide for fathers and having a child of any age is protective for mothers (Qin & Mortensen, 2003), we conducted sensitivity analysis with finer categories of parental status: having a child less than six years old, having a 6–18-year-old child, and having no children.

**Table 1:** Distribution of time at risk by background characteristics among men and women across marriage type, Sweden, 1991–2016

Characteristics	Men					Women				
	Sw-Sw^a^	Sw-Im	Im-Sw	Im-Inter-Im	Im-Intra-Im	Sw-Sw	Sw-Im	Im-Sw	Im-Inter-Im	Im-Intra-Im
*Person-Years*	31,329,020^b^	1,838,110	1,519,840	524,920	2,628,060	31,444,900	1,350,260	2,152,480	619,570	3,177,510
*Education*										
Prim-Second	72.25	69.07	63.74	59.82	65.41	69.44	65.19	61.02	58.22	66.16
Post-Second	26.70	30.29	33.16	33.04	26.55	29.98	34.51	36.78	34.73	25.15
Missing	1.05	0.64	3.10	5.68	8.04	0.58	0.30	2.20	7.05	8.69
*Income*										
Low	9.58	11.00	12.84	26.43	25.64	10.19	11.38	13.22	26.88	26.01
Medium	38.86	41.03	40.63	43.73	46.38	38.52	40.59	40.78	44.52	47.27
High	51.56	47.97	46.53	26.20	27.98	51.29	48.03	46.00	28.59	26.71
*Employment*										
Employed	95.68	94.87	92.67	90.42	90.55	93.68	92.09	91.46	89.85	90.43
Not employed	4.32	5.13	7.33	9.58	9.45	6.32	7.91	8.54	10.15	9.57
*Parental status*										
Child below 18	68.40	68.74	61.85	55.67	55.58	67.98	63.24	67.16	54.15	52.97
No child/adult child	31.60	31.26	38.15	44.33	44.42	32.02	36.76	32.84	45.85	47.03
*Country of birth*										
Western countries	–	–	67.00	29.35	25.65	–	–	59.45	26.04	22.95
Other European countries	–	–	14.15	23.92	32.76	–	–	17.58	31.68	30.52
All others	–	–	18.85	46.73	41.58	–	–	22.96	42.29	46.53

^a^Sw-Sw: Swedish – Swedish, Sw-Im: Swedish – Immigrant; Im-Sw: Immigrant – Swedish; Im-Inter-Im: Immigrant – Immigrant from different countries; Im-Intra-Im: Immigrant – Immigrant from the same country

^b^in 1000 person-years

## Results

In total, 6,249,727 married individuals aged 18 and older were included in our sample; 3,316,524 (53%) of them were men and 1,120,540 (18%) were immigrants. Over the period from 1991 to 2016, 18,116 deaths occurred due to suicide. As Table 1 demonstrates, for both genders the proportion of persons with post-secondary education was higher among those who entered intermarriages than among those who intramarried, whether they were of Swedish or immigrant origins. The percentage of high-income people was greater among Swedes than immigrants, irrespective of the immigration background of their spouse, and immigrants who married Swedes had higher incomes than immigrants married to other immigrants from either the same or another country. The percentage of unemployed men and women is lowest among Swedes irrespective of their partners’ immigration background, followed by immigrants in intermarriages with Swedish persons. Immigrants married to other foreign-born individuals—again whether from a different or the same country—were the most likely to be unemployed. Although the proportion of individuals with a minor in the household is high across all marriage types, immigrant men and women who were married to any other immigrant were the least likely to have a child below age 18. Intermarriages with native Swedes were most common among immigrants from Nordic and Western countries. Immigrant intermarriages and intramarriages were more frequent among immigrants from other than Nordic, Western and other European countries. All these patterns were apparent in both genders.

### Suicide and Marriage Type among Married Persons of Swedish and Immigrant Origins

Models 1 and 4 in Table 2 show unadjusted suicide mortality hazard ratios by marriage type for men and women, respectively.

**Table 2:** Hazard ratios for suicide death by marriage type in the total Swedish population, 1991–2016

	Men			Women		
	Model 1^a^	Model 2	Model 3	Model 4	Model 5	Model 6
	HR [95% CI]^b^	HR [95% CI]	HR [95% CI]	HR [95% CI]	HR [95% CI]	HR [95% CI]
*Marriage type (ref: Sw-Sw)*^*c*^						
Sw-Im	1.208^+++^	1.173^++^	1.171^++^	1.118	1.083	1.077
	[1.085,1.345]	[1.053,1.306]	[1.052,1.304]	[0.908,1.376]	[0.880,1.334]	[0.875,1.327]
Im-Sw	1.090	1.04	1.04	1.622^+++^	1.526^+++^	1.481^+++^
	[0.960,1.237]	[0.916,1.181]	[0.916,1.181]	[1.409,1.867]	[1.325,1.758]	[1.285,1.707]
Im-Inter-Im	0.994	0.805	0.805	1	0.789	0.771
	[0.790,1.251]	[0.638,1.014]	[0.638,1.014]	[0.708,1.413]	[0.557,1.117]	[0.544,1.091]
Im-Intra-Im	0.861^++^	0.673^+++^	0.674^+++^	0.838^+^	0.634^+++^	0.639^+++^
	[0.770,0.962]	[0.600,0.755]	[0.601,0.756]	[0.708,0.991]	[0.533,0.755]	[0.537,0.760]
*Education (ref: primary or secondary)*						
Post-Secondary		0.767^+++^	0.768^+++^		0.796^+++^	0.809^+++^
		[0.718,0.820]	[0.719,0.821]		[0.721,0.879]	[0.732,0.893]
Missing		1.123	1.122		1.455^+^	1.430^+^
		[0.952,1.324]	[0.951,1.323]		[1.087,1.947]	[1.068,1.914]
*Income (ref: medium)*						
Low		1.537^+++^	1.535^+++^		1.401^+++^	1.380^+++^
		[1.430,1.651]	[1.429,1.650]		[1.252,1.568]	[1.233,1.545]
High		0.601^+++^	0.602^+++^		0.556^+++^	0.572^+++^
		[0.564,0.639]	[0.565,0.640]		[0.506,0.612]	[0.520,0.629]
*Employment status (ref: employed)*						
Not employed		1.264^+++^	1.264^+++^		1.017	1.019
		[1.134,1.408]	[1.134,1.409]		[0.865,1.195]	[0.867,1.198]
*Parental status (ref: having no or only adult children)*						
Having a minor child			0.963			0.606^+++^
			[0.888,1.044]			[0.532,0.690]
N^b^	2,635,152			2,723,758		
Nr. deaths	6229			2549		

^a^Model 1: Marriage type; Model 2: Model 1 + socioeconomic characteristics; Model 3: Model 2 + having a child under 18

^b^Hazard ratio [95% Confidence Interval], N–number of subjects

^c^Sw-Sw: Swedish–Swedish, Sw-Im: Swedish–Immigrant; Im-Sw: Immigrant–Swedish; Im-Inter-Im: Immigrant–Immigrant from different country of birth; Im-Intra-Im: Immigrant–Immigrant from the same country of birth

+ *p* < 0.05; + + *p* < 0.01; + + + *p* < 0.001

Compared to men in Swedish intramarriages, native-born men married to immigrants had 21% elevated hazard of suicide death (Hazard Ratio [HR] = 1.21, 95% Confidence Interval [CI]: 1.09, 1.35). The hazard of suicide mortality among immigrant men married to natives and to other immigrants from different countries was similar to that of Swedish men married to natives. In contrast, male immigrants married to another immigrant from the same country of birth had about 14% (HR = 0.86, 95% CI: 0.77, 0.96) lower hazard of suicide death relative to men in Swedish intramarriages.

The hazard of suicide death among Swedish women married to immigrants was similar to that of the reference group, whereas immigrant women married to Swedes had about 62% (HR = 1.62, 95% CI: 1.41, 1.87) elevated mortality hazard compared to women in Swedish intramarriages. In line with the patterns observed in the male study population, the hazard ratio among women in immigrant intermarriages was similar to the comparison group, while being married to another immigrant from the same country was associated with a lower mortality hazard relative to Swedish women married to native persons (HR = 0.84, 95% CI: 0.71, 0.99).

In Models 2 and 5, we tested whether socioeconomic characteristics account for suicide mortality differences across inter- and intra-marriage groups. The results show that having post-secondary education and high-income predict a lower mortality hazard among both men and women, while employment status appears to be an important predictor of suicide death only among men. When socioeconomic status is included in Model 2, the hazard of suicide death was slightly attenuated for native-born men in an intermarriage (HR = 1.17, 95% CI: 1.05, 1.31), and it decreased noticeably among immigrant men in intramarriage unions (HR = 0.67, 95% CI: 0.60, 0.76) relative to Swedish intramarriages. No substantive changes in the mortality hazard were observed among immigrant men in Swedish and immigrant intermarriages when socioeconomic characteristics were included in Model 2. Accounting for differences in socioeconomic characteristics across marriage type, the hazard of suicide death among immigrant women married to Swedish men was slightly attenuated, but it remained significantly higher compared to Swedish intramarriages. As it does for men, including socioeconomic characteristics in Model 5 resulted in a further reduction of mortality hazard for immigrant women in co-ethnic unions relative to women in a Swedish intramarriage.

In Models 3 and 6 we examined whether having a minor child confounds the relationship between marriage type and suicide death. Having a minor child is independently related to about 39% lower suicide hazard for women (HR = 0.61, 95% CI: 0.53, 0.69), but not for men. Accounting for children only slightly attenuates the increased hazard of mortality for immigrant women married to Swedes compared to their female peers in Swedish intramarriages.

### Suicide and Marriage Type among Married Persons of Immigrant Origins

In further analysis we considered only the immigrant population, with immigrant intramarriages as the reference category. First, we ran sex-specific models that included marriage type as well as socioeconomic characteristics and parental status (Table 3, Models 1 and 3). In Models 2 and 4, we added country of birth categories to assess whether immigrant-specific characteristics account for survival differences across inter- and intra-marriage groups among men and women, respectively.

**Table 3:** Hazard ratios for suicide death by marriage type in the immigrant population, Sweden, 1991–2016

	Men		Women	
	Model 1^a^	Model 2	Model 3	Model 4
	HR [95% CI]^b^	HR [95% CI]	HR [95% CI]	HR [95% CI]
*Marriage type (ref: Im-Intra-Im)*^*c*^				
Im-Sw	1.338^+++^	1.162	2.129^+++^	1.965^+++^
	[1.130,1.585]	[0.971,1.390]	[1.709,2.651]	[1.564,2.469]
Im-Inter-Im	1.195	1.209	1.229	1.201
	[0.929,1.539]	[0.939,1.557]	[0.842,1.795]	[0.822,1.754]
*Education (ref: primary or secondary)*				
Post-Second	0.677^+++^	0.708^+++^	0.722^++^	0.731^+^
	[0.558,0.822]	[0.582,0.860]	[0.565,0.924]	[0.571,0.937]
Missing	0.711	0.729	1.367	1.418
	[0.501,1.009]	[0.514,1.035]	[0.913,2.048]	[0.946,2.127]
*Income (ref: medium)*				
Low	1.312^++^	1.399^+++^	1.158	1.216
	[1.077,1.599]	[1.147,1.706]	[0.895,1.498]	[0.939,1.575]
High	0.851	0.808^+^	0.720^++^	0.684^++^
	[0.703,1.031]	[0.667,0.980]	[0.564,0.921]	[0.534,0.876]
*Employment status (ref: employed)*				
Not employed	0.912	0.95	0.848	0.847
	[0.667,1.247]	[0.695,1.300]	[0.570,1.262]	[0.569,1.261]
*Parental status (ref: having no or only adult children)*				
Having a minor child	0.842	0.928	0.573^+++^	0.599^+++^
	[0.680,1.042]	[0.746,1.154]	[0.430,0.765]	[0.448,0.801]
*Country of birth (ref: Nordic & Western countries)*^§^				
Other European		0.855		0.987
		[0.705,1.038]		[0.770,1.266]
All other countries		0.491		0.497
		[0.389, 0.619]		[0.367, 0.675]
N^b^	444,290		559,101	
Nr. deaths	660		397	

^a^Model 1: Marriage type; Model 2: Model 1 + socioeconomic characteristics; Model 3: Model 2 + having a child under 18

^b^Hazard ratio [95% confidence interval], N – number of subjects

^c^Sw-Sw: Swedish–Swedish, Sw-Im: Swedish–Immigrant; Im-Sw: Immigrant–Swedish; Im-Inter-Im: Immigrant–Immigrant from different country of birth; Im-Intra-Im: Immigrant–Immigrant from the same country of birth

+ *p* < 0.05; ++ *p* < 0.01; +++ *p* < 0.001

Model 1 of Table 3 shows that, when socioeconomic and parental status were held constant, the hazard of suicide was 34% higher among immigrant men married to Swedes than men in immigrant intramarriages (HR = 1.34, 95% CI: 1.13, 1.59). No mortality differentials were observed among immigrant men married to other immigrants from either different or the same country of birth. When country of birth was included (Model 2), the elevated mortality hazard among immigrant men married to Swedes relative to their peers in immigrant intramarriages was completely attenuated. As previously, the hazard of suicide death was similar among immigrants married to their peers from both different and the same countries. In Model 3 of Table 3, which was adjusted for differences in socioeconomic and parental status, the hazard of suicide death among immigrant women married to Swedes was about two times higher than for immigrant women in intramarriages (HR = 2.13, 95% CI: 1.71, 2.65). As in the male sample, no mortality differentials were observed among immigrant women married to immigrants from a different or the same country. When country of birth was included in Model 4, the elevated hazard of mortality among immigrant women married to Swedes was slightly reduced but remained almost twice as high as for the reference group (HR = 1.96, 95% CI: 1.56, 2.47). The hazard of suicide mortality among immigrant women married to immigrants from different countries remained almost unchanged.

These analyses also show that both male and female immigrants from non-Western countries have lower hazard of suicide mortality relative to immigrants from high-income countries (Nordic countries, Western Europe, North America, Australia, and New Zealand). Immigrant men and women from other European countries have similar risk of suicide death as their peers from high-income countries.

Prior work suggests that younger age at immigration is linked to greater integration in host societies (Aslund et al., 2009; Söhn, 2011). Individuals who immigrate when they are young may thus be more likely both to marry a Swede and to resemble native-born persons in their suicide behaviors than those who arrived in adulthood. To advance our understanding of the mechanisms underlying the elevated suicide mortality of immigrants married to Swedes, we repeated the regression analyses among immigrants that arrived in Sweden before and after age 18 separately. Table 4 shows that holding country of birth and socioeconomic and parental statuses constant, there was no relationship between the immigration background of married couples and hazard of suicide mortality among men and women who arrived to Sweden before age 18.

**Table 4:** Hazard ratios for suicide death by marriage type in the immigrant population by age at immigration, Sweden, 1991–2016

	18 years or below at immigration		18 + years at immigration	
	Men	Women	Men	Women
	HR [95% CI]^a^	HR [95% CI]	HR [95% CI]	HR [95% CI]
*Marriage type (ref: Im-Intra-Im)*^*b*^				
Im-Sw	1.232	1.885	1.176	2.002^+++^
	[0.725,2.093]	[0.967,3.674]	[0.966,1.432]	[1.567,2.558]
Im-Inter-Im	1.322	0.970	1.188	1.223
	[0.636,2.748]	[0.312,3.017]	[0.905,1.558]	[0.818,1.829]
*Education (ref: primary or secondary)*				
Post-Second	0.619	0.526	0.726^++^	0.773
	[0.352,1.088]	[0.262,1.060]	[0.589,0.894]	[0.591,1.010]
Missing^c^	–	–	0.771	1.550^+^
			[0.542,1.097]	[1.027,2.340]
*Income (ref: medium)*				
Low	2.230^++^	2.296^+^	1.330^++^	1.103
	[1.283,3.876]	[1.218,4.326]	[1.076,1.645]	[0.832,1.462]
High	0.692	0.435^++^	0.841	0.754^+^
	[0.428,1.119]	[0.238,0.795]	[0.681,1.038]	[0.575,0.989]
*Employment status (ref: employed)*				
Not employed	1.248	0.998	0.887	0.795
	[0.637,2.444]	[0.448,2.222]	[0.622,1.266]	[0.502,1.260]
*Parental status (ref: having no or older children)*				
Having a minor child	0.692	0.429^++^	0.978	0.643^++^
	[0.414,1.157]	[0.226,0.816]	[0.770,1.243]	[0.465,0.889]
*Country of birth (ref: western countries)*				
Other European	0.896	0.746	0.842	1.001
	[0.498,1.613]	[0.326,1.705]	[0.685,1.035]	[0.767,1.305]
All others	0.708	0.927	0.463^+++^	0.453^+++^
	[0.369,1.360]	[0.440,1.953]	[0.361,0.594]	[0.324,0.634]
N^a^	57,632	74,157	386,658	484,944
Nr. deaths	96	64	564	333

^a^Hazard ratio [95% Confidence Interval], *N*–number of subjects

^b^Im-Sw: Immigrant–Swedish; Im-Inter-Im: Immigrant–Immigrant from different country of birth; Im-Intra-Im: Immigrant–Immigrant from the same country of birth

^c^No missing on education for those arriving as minors because they get education in Sweden

+ *p* < 0.05; +  + *p* < 0.01; +  +  + *p* < 0.001

In a second set of exploratory analyses, we only include those immigrants who arrived as adults (after age of 18) to identify whether any of the patterns we previously found were driven by immigrants from specific regions. We ran the final models (adjusted for socioeconomic and parental status) by gender for each of three groups of countries separately. The analyses indicated that immigrant women from all region-specific groups except for Nordic and Western European women married to Swedes have higher hazard of suicide death relative to women in co-ethnic marriages (Table 5). Specifically, being married to a Swede increased the hazard for Eastern European more than two-fold (HR = 2.57, 95% CI: 1.68, 3.93) and for women from other Non-Western countries almost four-fold (HR = 3.86, 95% CI: 2.19, 6.80). Marriage type was unrelated to suicide mortality among immigrant men from Eastern European, Nordic and Western countries but immigrant men from other Non-Western countries had 2.4 times higher hazard of suicide death compared to co-ethnically married peers (HR = 2.38, 95% CI: 1.49, 3.79). For men in all other immigrant groups the hazard of suicide death did not differ by marriage type.

**Table 5:** Hazard ratios for suicide death by marriage type and by country of birth in the immigrant population, Sweden, 1991–2016

	Women			Men		
	Eastern Europe	All others non-western	Nordic & Western countries^a^	Eastern Europe	All others non-western	Nordic & Western countries^a^
	HR [95%CI]^b^	HR [95%CI]	HR [95%CI]	HR [95%CI]^b^	HR [95%CI]	HR [95%CI]
*Marriage type (ref.: Im-Intra-Im)*^*c*^						
Im-Sw	2.569^+++^	3.863^+++^	1.392	1.080	2.378^+++^	0.949
	[1.681,3.926]	[2.194,6.801]	[1.000,1.938]	[0.683,1.708]	[1.493,3.788]	[0.744,1.211]
Im-Inter-Im	1.654	1.123	0.998	1.516	1.496	0.789
	[0.908,3.012]	[0.433,2.914]	[0.509,1.956]	[0.970,2.367]	[0.883,2.534]	[0.496,1.255]
*Education (ref: primary or secondary)*						
Post-Second	0.880	1.308	0.515^++^	0.638^+^	0.842	0.706^+^
	[0.569,1.362]	[0.741,2.311]	[0.327,0.809]	[0.429,0.949]	[0.554,1.277]	[0.516,0.965]
Missing	1.639	2.082	1.318	0.962	0.793	0.578
	[0.778,3.453]	[0.967,4.484]	[0.665,2.614]	[0.542,1.708]	[0.350,1.798]	[0.329,1.015]
*Income (ref: medium)*						
Low	0.952	1.326	1.139	1.643^+^	0.816	1.425^+^
	[0.571,1.585]	[0.724,2.428]	[0.751,1.726]	[1.101,2.451]	[0.507,1.312]	[1.056,1.922]
High	0.838	0.747	0.694	1.182	0.622	0.755
	[0.529,1.327]	[0.369,1.516]	[0.473,1.018]	[0.798,1.751]	[0.379,1.022]	[0.564,1.011]
	0.952	1.326	1.139	1.643^+^	0.816	1.425^+^
*Employment status (ref: employed)*						
Not employed	0.844	1.036	0.632	0.540	0.900	1.158
	[0.405,1.758]	[0.439,2.444]	[0.277,1.442]	[0.236,1.235]	[0.489,1.658]	[0.691,1.942]
*Parental status (ref: having no or older children)*						
Having a minor	0.591	0.920	0.651	1.109	0.786	
	[0.326,1.071]	[0.518,1.633]	[0.373,1.135]	[0.700,1.757]	[0.507,1.220]	
N^b^	121,014	229,930	134,000	89,480	174,348	122,830
Nr. deaths	107	60	166	162	108	294

^a^Nordic and Western European countries, North America (USA and Canada), Australia, and New Zealand

^b^Hazard ratio [95% confidence interval], *N*–number of subjects

^c^Im-Sw: Immigrant–Swedish; Im-Inter-Im: Immigrant–Immigrant from different country of birth; Im-Intra-Im: Immigrant–Immigrant from the same country of birth

+ *p* < 0.05; ++ *p* < 0.01; +++ *p* < 0.0

We also performed sensitivity analysis with all-cause mortality in the total and immigrant populations (Supplementary Tables 1 and 2, respectively). If the patterns observed for all-cause mortality are the same as in the analysis for suicide mortality, this would support the hypothesis that selection into specific marriage type and/or health selection among immigrants explains the elevated hazard of suicide deaths among intermarried Swedish men and immigrant women and intramarried immigrants. Supplementary Table 1 shows that, as with suicide mortality, Swedish men married to immigrant women have a higher HR of all-cause mortality, although the point estimates are substantially lower than what we observed for suicide mortality. In contrast to our findings for suicide mortality, we did not find an elevated hazard for all-cause mortality among immigrant women married to native men. The hazard of all-cause death is higher among Swedish women married to immigrant men relative to their peers in Swedish intramarriages. Additionally, immigrant women married to immigrant men from a different or the same country have lower mortality HRs, while in the analysis of suicide death this pattern was apparent only for intramarried immigrant women.

HRs for all-cause mortality in the immigrant population are shown in Supplementary Table 2. In contrast to the main analysis, immigrant men married to Swedish women have a lower hazard for all-cause mortality relative to their co-ethnically married peers. Although immigrant women married to Swedes had an elevated all-cause mortality hazard in the model adjusted for socioeconomic characteristics, the point estimate was substantially lower than the HR for suicide mortality and changed to not significant after adjusting for the region of birth. As in the main analysis, the hazard of all-cause mortality among immigrant men and women married to immigrants from the same or different countries was similar.

Overall, our sensitivity analysis suggests that although there might be a negative selection into marriage among Swedish men and women who marry immigrants, and a positive selection with respect to health among immigrant women, these selection processes can only partially explain the elevated suicide HRs for Swedish-immigrant intermarried partners. They thus provide additional support for our hypothesis that marital strain in inter-ethnically married couples contributes to increased suicide mortality.

## Discussion

The present study took a first step toward investigating the effects of inter- and intra-ethnic marriages on suicide mortality among immigrant and native-born persons. We showed that both partners in marriages between native-born Swedish men and female immigrants had a substantially elevated hazard of suicide death in comparison to intramarried Swedes. Additionally, immigrant men and women who were married to immigrants from their same origin country had a markedly reduced hazard of suicide relative to intramarried Swedes. These findings partially support our hypothesis that intermarried persons would experience greater suicide risk than intramarried persons, informed by research suggesting that these relationships have higher instability and rates of marital discord. They also support our hypothesis that intramarried immigrants, who share migration experiences and are on average healthier than native-born persons, would have the lowest suicide risk. While suicide rates and gender differences in suicide rates vary across nations in ways that may also contribute to these patterns, within-immigrant analyses accounting for region-of-origin help to account for these possibilities.

One explanation for the elevated suicide hazard found for both partners in intermarriages between immigrant women and native-born men is that these relationships may be characterized by cultural conflict and instability. Studies in the US and Europe provide compelling evidence that interethnic unions are less stable than same-ethnic marriages, due at least in part to differences in shared values, cultural and relationship norms, and communication styles, all of which can increase misunderstandings and diminish the quality of marital relationships (Dribe & Lundh, 2012; Hohmann-Marriott & Amato, 2008; Kalmijn et al., 2005; Milewski & Kulu, 2014). Our analyses further reveal that the elevated suicide risk associated with intermarriage among immigrants is limited to those from non-Western countries; suicide rates for intermarried immigrants from Western countries are similar to those found among intramarried immigrants. This finding resonates with earlier research indicating that marital discord increases along with dissimilarity in cultural backgrounds between spouses in intermarriages (Dribe & Lundh, 2011). However, we did not find a similar elevated risk of suicide mortality among those in intermarriages between Swedish women and immigrant men.

Our finding of an elevated risk of suicide mortality among immigrant women married to Swedish men but not immigrant men married to native-born women may be related to the characteristics of intermarriages. Drawing on the status exchange hypothesis originally proposed by Davis (1941) and Merton (1941), Kalmijn and van Tubergen (2006) showed that marrying down in terms of education was more likely among intermarried couples than among intramarried couples (including both native-native and immigrant-immigrant intramarriages). Specifically, immigrant men were more likely to marry down in terms of education when they married a native-born woman compared to when they married an immigrant woman, i.e., to trade-off their higher socioeconomic status with the prestige of marrying a native woman (Kalmijn & van Tubergen, 2006). Also, prior studies in Germany and Sweden revealed that immigrant men marrying native women had higher levels of life satisfaction and earning potential prior to the union formation (Dribe & Nystedt, 2011; Potarca & Bernardi, 2021), suggesting they are better off than peers who intramarry with respect to their mental health and economic and human capital. Since the traditional male-breadwinner model is still the most common form of partnership, a husband’s socioeconomic position may be seen as more important than wife’s socioeconomic position. Hence, the exchange of higher socioeconomic position with a high native prestige may be less beneficial for immigrant women––Swedish men intermarriages, and these partnerships may experience greater marital discord than intermarriages involving immigrant men and Swedish women. Binational marriages in which immigrant women marry down to native Swedish men may be especially likely to suffer from the asymmetric family power relations that are still common in Sweden and other European countries, where men enjoy the status of breadwinner and women are expected to be homemakers (Hederos et al., 2017; Riaño, 2011; Riaño & Baghdadi, 2007). Skilled female immigrants from non-European countries may struggle to conform to these expectations and adjust their pre-immigration vision of gender equality in European countries, creating marital conflict. This possibility resonates with our findings of especially high levels of education among immigrants who are married to Swedes, and that adjusting for socioeconomic status completely attenuated the increased hazard of suicide death among immigrant men who were intermarried to native Swedes, but only slightly among immigrant women with Swedish partners. Additionally, studies show that immigrant wives are especially likely to be dependent on their native husbands not only economically, but also legally (Dribe & Lundh, 2011; Riano et al., 2015). US-based research indicates that depression levels are strongly affected by perceptions of marital equity among both men and women (Glass & Fujimoto, 1994). Inequalities in marital power and the household division of labor may thus have negative effects on both partners in intermarriages involving female immigrants and native-born men (Wanic & Kulik, 2011).

Immigrating at younger ages predicts better social and economic integration (Bleakley & Chin, 2010). To further explore the potential role of cultural differences and material inequalities in relationships with native-born spouses in explaining some intermarried immigrants’ greater risk for suicide, we split the immigrant sample between those that immigrated in childhood and after age 18. These analyses revealed that the immigration background of one’s spouse was unrelated to suicide mortality among immigrants who moved to Sweden when they were children. Intermarrying with a native-born person was positively linked to suicide mortality only for immigrant women who moved to the country after age 18. We believe these findings highlight that the greater social integration and resources of those who immigrate in childhood may attenuate any disadvantages associated with intermarrying.

Selection mechanisms may help to explain why we find the lowest suicide hazard among intramarried immigrants, since a large body of research suggests that immigrants are positively selected in terms of their health. Additionally, although they do not derive the social integration benefits of having a native spouse, intramarried immigrants share the same culture, native language, and immigration experiences, which may cumulatively reduce marital discord.

Our study revealed a clear gendered pattern in the role of employment and parental statuses in predicting suicide death: being employed is protective for men but not for women, and having minor children is protective for women but not for men. While these findings highlight the traditional gendered division of labor even in a country that has the highest gender equality index across European countries in 2020 (Barbieri et al., 2022), they also point toward potential avenues for strategies tailored to prevent mental health problems among men and women.

Using register data covering the whole Swedish population, we were able to explore whether country-specific characteristics and social integration underpin the relationship between marriage type and suicide mortality, which would not be possible in many studies due to the low occurrence of this event. However, register data also have limits. Since suicide is considered to be a largely preventable public health problem (Word Health Organization, 2004), it is important to take into account previous mental health problems and treatment in suicide research. Unfortunately, this information is not available in our data. Additionally, we were unable to include cohabiting partnerships because partnerships without children cannot be identified before 2011. Future studies should explore whether mental health differences also exist between cohabitating couples with different immigration backgrounds. Examining the role of partner characteristics in explaining intermarriage-suicide association is another important avenue for further studies. A recent study in Sweden showed that odds of intermarriage, especially among Swedish men and immigrant women, depend on marital age difference being highest in age-hypergamous unions (Elwert, 2020). Such detailed analyses of health benefits by marriage type and marital age difference need to focus on a more prevalent mental health outcome than suicide death to obtain meaningful results. More broadly, research that focuses on the role of partners’ characteristics and experiences of relationship quality at the micro-level and neighborhood characteristics at the macro-level represents another promising way to expand knowledge about the health and integration of the growing population of immigrants in Nordic nations. A last important limitation is that while we were able to distinguish among immigrants originating from certain regions, the data could not support country-specific analyses. Suicide rates also vary within these heterogenous regional groups in ways that we were unable to account for in this analysis and should be investigated in future.

Overall, our findings provide compelling preliminary evidence that some individuals in intermarriages between immigrants and native-born persons are at increased risk of suicide mortality. They thus highlight the possibility that stressors introduced by intermarrying, such as cultural conflict, marital discord, and relationship inequality, have long-term consequences for individuals’ mental health and well-being.

### Supplementary Information

Below is the link to the electronic supplementary material.

Supplementary file1 (DOCX 69 KB)

## Data Availability

The study utilized the Swedish register data which includes personal data (e.g., causes of death) at the individual level. These data can be made available in Supporting Information files or in a data repository because of ethical restrictions imposed by the Swedish Data Protection Agency (Stockholm, Sweden) and the European Data Protection Law.
