# Supplementary material for: Is it Better to Intermarry? Immigration Background of Married Couples and Suicide Risk Among Native-Born and Migrant Persons in Sweden
Source: Eur J Popul. 2023 Mar 8;39(1):8. doi: 10.1007/s10680-023-09650-x (PMC9995640; doi:10.1007/s10680-023-09650-x)
Supplement: Supplementary file 1 — Supplementary file1 (DOCX 69 KB) [file 10680_2023_9650_MOESM1_ESM.docx]

**Supplementary Table 1. All-cause mortality hazard ratios by marriage type in the total Swedish population, 1991–2016**

|  | **Men** |  |  | **Women** |  |  |
| --- | --- | --- | --- | --- | --- | --- |
|  | **Model 1^a^** | **Model 2** | **Model 3** | **Model 4** | **Model 5** | **Model 6** |
|  | HR  [95% CI]^b^ | HR  [95% CI] | HR  [95% CI] | HR  [95% CI] | HR  [95% CI] | HR  [95% CI] |
| **Marriage type (ref: Sw-Sw)**^c^ | | |  |  |  |  |
| Sw-Im | 1.040^+++^ | 1.054^+++^ | 1.055^+++^ | 1.037^++^ | 1.066^+++^ | 1.065^+++^ |
|  | [1.027,1.054] | [1.041,1.068] | [1.042,1.069] | [1.012,1.062] | [1.040,1.092] | [1.040,1.092] |
| Im-Sw | 0.958^+++^ | 0.984 | 0.985 | 0.987 | 0.955 | 0.992 |
|  | [0.942,0.974] | [0.967,1.000] | [0.968,1.001] | [0.968,1.006] | [0.976,1.014] | [0.973,1.012] |
| Im-Inter-Im | 1.014 | 1.000 | 1.004 | 0.918^+++^ | 0.888^+++^ | 0.885^+++^ |
|  | [0.893,1.054] | [0.970,1.031] | [0.974,1.035] | [0.875,0.963] | [0.846,0.932] | [0.844,0.929] |
| Im-Intra-Im | 1.081^++^ | 1.004 | 1.007 | 1.004 | 0.908^+++^ | 0.909^+++^ |
|  | [1.066,1.095] | [0.990,1.018] | [0.993,1.21] | [0.984,1.024] | [0.890,0.927] | [0.891,0.927] |
| **Education (ref: Primary or secondary)** | | |  |  |  |  |
| Post-Secondary | | 0.800^+++^ | 0.802^+++^ |  | 0.719^+++^ | 0.723^+++^ |
|  |  | [0.794,0.807] | [0.795,0.809] |  | [0.709,0.728] | [0.714,0.733] |
| Missing |  | 1.123^+++^ | 1.123^+++^ |  | 1.187^+++^ | 1.187^+++^ |
|  |  | [1.112,1.134] | [1.112,1.134] |  | [1.168,1.207] | [1.168,1.207] |
| **Income (ref: Medium)** | | |  |  |  |  |
| Low |  | 1.163^+++^ | 1.163^+++^ |  | 1.167^+++^ | 1.167^+++^ |
|  |  | [1.156,1.170] | [1.156,1.170] |  | [1.156,1.177] | [1.156,1.177] |
| High |  | 0.761^+++^ | 0.762^+++^ |  | 0.738^+++^ | 0.741^+++^ |
|  |  | [0.754,0.767] | [0.755,0.769] |  | [0.729,0.748] | [0.732,0.751] |
| **Employment status (ref: Employed)** | | |  |  |  |  |
| Not employed |  | 0.938^+++^ | 0.938^+++^ |  | 0.744^+++^ | 0.744^+++^ |
|  |  | [0.910,0.967] | [0.911,0.967] |  | [0.717,0.773] | [0.717,0.772] |
| **Parental status (ref: Having no or only adult children)** | | | |  |  |  |
| Having a child below 6 | |  | 0.718^+++^ |  |  | 0.507^+++^ |
|  |  |  | [0.684,0.754] |  |  | [0.473,0.544] |
| Having a 6-18 years old child | | | 0.820^+++^ |  |  | 0.734^+++^ |
|  |  |  | [0.800, 0.840] | |  | [0.709,0.759] |
| N^b^ | 2,635,152 |  |  | 2,723,758 |  |  |
| Nr. deaths | 6229 |  |  | 2549 |  |  |

a: Model 1: Marriage type; Model 2: Model 1+ socioeconomic characteristics; Model 3: Model 2 + parental status

b: Hazard ratio [95% Confidence Interval], N – number of subjects

c: Sw-Sw: Swedish – Swedish, Sw-Im: Swedish – Immigrant; Im-Sw: Immigrant – Swedish; Im-Inter-Im: Immigrant – Immigrant from different country of birth; Im-Intra-Im: Immigrant – Immigrant from the same country of birth

+ p-value <0.05; ++ p-value < 0.01; +++ p-value <0.001

**Supplementary Table 2. All-cause mortality hazard ratios by marriage type in the immigrant population, Sweden, 1991–2016**

|  | **Men** |  | **Women** |  |
| --- | --- | --- | --- | --- |
|  | **Model 1^a^** | **Model 2** | **Model 3** | **Model 4** |
|  | HR  [95% CI]**^b^** | HR  [95% CI] | HR  [95% CI] | HR  [95% CI] |
| **Marriage type (ref: Im-Intra-Im)**^c^ | | |  |  |
| Im-Sw | 0.961^+++^ | 0.928^+++^ | 1.061^+++^ | 1.016 |
|  | [0.940,0,982] | [0.907,0.949] | [1.032,1.092] | [0.986,1.046] |
| Im-Inter-Im | 0.997 | 0.992 | 0.964 | 0.954 |
|  | [0.964,1.030] | [0.959,1.025] | [0.915,0.016] | [0.905,1.005] |
| **Education (ref: Primary or secondary)** | | |  |  |
| Post-Second | 0.753^+++^ | 0.770^+++^ | 0.734^++^ | 0.748^+++^ |
|  | [0.732,0.755] | [0.748,0.792] | [0.704,0.765] | [0.717,0.779] |
| Missing | 0.971 | 1.016 | 1.052^+^ | 1.110^+++^ |
|  | [0.941,1.003] | [0.984,1.049] | [1.004,1.102] | [1.058,1.163] |
| **Income (ref: Medium)** | | |  |  |
| Low | 1.128^+++^ | 1.164^+++^ | 1.154^+++^ | 1.185^+++^ |
|  | [1.102,1.154] | [1.137,1.191] | [1.120,1.189] | [1.150,1.222] |
| High | 0.763^+++^ | 0.760^+++^ | 0.745^++^ | 0.736^+++^ |
|  | [0.740,0.787] | [0.736,0.784] | [0.714,0.777] | [0.705,0.768] |
| **Employment status (ref: Employed)** | | |  |  |
| Unemployed | 0.832^+++^ | 0.846^+++^ | 0.732^+++^ | 0.736^+++^ |
|  | [0.768,0.902] | [0.780,0.917] | [0.660,0.812] | [0.664,0.816] |
| **Parental status (ref: Having no or only adult children)** | | |  |  |
| Having a  child below 6 | 0.750^+++^ | 0.810^+++^ | 0.535^+++^ | 0.555^+++^ |
|  | [0.678,0.829] | [0.732,0.897] | [0.461,0.621] | [0.478,0.644] |
| Having a 6-18 years old  Child | 0.847^+++^ | 0.911^++^ | 0.728^+++^ | 0.757^+++^ |
|  | [0.801,0.897] | [0.860,0.965] | [0.670,0.792] | [0.696,0.823] |
| **Country of birth (ref: Nordic & Western countries)**^§^ | | |  |  |
| Other European |  | 0.991 |  | 0.927^+++^ |
|  |  | [0.968,1.015] |  | [0.896,0.959] |
| All other countries |  | 0.736^+++^ |  | 0.719^+++^ |
|  |  | [0.712, 0.761] |  | [0.685, 0.755] |
|  |  | 0.991 |  | 0.927^+++^ |
| N^b^ | 444,290 |  | 559,101 |  |
| Nr. deaths | 40,612 |  | 22,560 |  |

a: Model 1: Marriage type, education, income, and employment and parental status; Model 2: Model 1 + country of birth

b: Hazard ratio (95% Confidence Interval), N – number of subjects

c:Im-Sw: Immigrant – Swedish; Im-Inter-Im: Immigrant – Immigrant from different country of birth; Im-Intra-Im: Immigrant – Immigrant from the same country of birth

Nordic, Western European, and North American countries, Australia, New Zealand

**Supplementary Table 3. Hazard ratios for death by suicide by marriage type in the total Swedish population, 1991–2016**

|  | **Men** |  |  | **Women** |  |  |
| --- | --- | --- | --- | --- | --- | --- |
|  | **Model 1^a^** | **Model 2** | **Model 3** | **Model 4** | **Model 5** | **Model 6** |
|  | HR  [95% CI]^b^ | HR  [95% CI] | HR  [95% CI] | HR  [95% CI] | HR  [95% CI] | HR  [95% CI] |
| **Marriage type (ref: Sw-Sw)**^c^ | | |  |  |  |  |
| Sw-Im | 1.208^+++^ | 1.173^++^ | 1.174^++^ | 1.118 | 1.083 | 1.079 |
|  | [1.085,1.345] | [1.053,1.306] | [1.054,1.307] | [0.908,1.376] | [0.880,1.334] | [0.876,1.328] |
| Im-Sw | 1.090 | 1.04 | 1.041 | 1.622^+++^ | 1.526^+++^ | 1.480^+++^ |
|  | [0.960,1.237] | [0.916,1.181] | [0.917,1.182] | [1.409,1.867] | [1.325,1.758] | [1.284,1.705] |
| Im-Inter-Im | 0.994 | 0.805 | 0.807 | 1 | 0.789 | 0.770 |
|  | [0.790,1.251] | [0.638,1.014] | [0.640,1.017] | [0.708,1.413] | [0.557,1.117] | [0.544,1.091] |
| Im-Intra-Im | 0.861^++^ | 0.673^+++^ | 0.674^+++^ | 0.838^+^ | 0.634^+++^ | 0.637^+++^ |
|  | [0.770,0.962] | [0.600,0.755] | [0.601,0.756] | [0.708,0.991] | [0.533,0.755] | [0.535,0.759] |
| **Education (ref: Primary or secondary)** | | |  |  |  |  |
| Post-Secondary |  | 0.767^+++^ | 0.771^+++^ |  | 0.796^+++^ | 0.814^+++^ |
|  |  | [0.718,0.820] | [0.721,0.824] |  | [0.721,0.879] | [0.737,0.898] |
| Missing |  | 1.123 | 1.122 |  | 1.455^+^ | 1.425^+^ |
|  |  | [0.952,1.324] | [0.951,1.323] |  | [1.087,1.947] | [1.065,1.907] |
| **Income (ref: Medium)** | | |  |  |  |  |
| Low |  | 1.537^+++^ | 1.534^+++^ |  | 1.401^+++^ | 1.375^+++^ |
|  |  | [1.430,1.651] | [1.428,1.648] |  | [1.252,1.568] | [1.229,1.540] |
| High |  | 0.601^+++^ | 0.600^+++^ |  | 0.556^+++^ | 0.570^+++^ |
|  |  | [0.564,0.639] | [0.563,0.638] |  | [0.506,0.612] | [0.518,0.627] |
| **Employment status (ref: Employed)** | | |  |  |  |  |
| Unemployed |  | 1.264^+++^ | 1.264^+++^ |  | 1.017 | 1.018 |
|  |  | [1.134,1.408] | [1.134,1.408] |  | [0.865,1.195] | [0.866,1.197] |
| **Parental status (ref: Having no or only adult children)** | | | |  |  |  |
| Having a child <6 years old | |  | 0.857^+++^ |  |  | 0.448^+++^ |
|  |  |  | [0.758,0.970] |  |  | [0.361,0.550] |
| Having a 6-18 year old child | |  | 0.997 |  |  | 0.660^+++^ |
|  |  |  | [0.916,1.085] |  |  | [0.577,0.755] |
| N^b^ | 2,635,152 |  |  | 2,723,758 |  |  |
| Nr. deaths | 6229 |  |  | 2549 |  |  |

a: Model 1: Marriage type; Model 2: Model 1+ socioeconomic characteristics; Model 3: Model 2 + parental status

b: Hazard ratio [95% Confidence Interval], N – number of subjects

c: Sw-Sw: Swedish – Swedish, Sw-Im: Swedish – Immigrant; Im-Sw: Immigrant – Swedish; Im-Inter-Im: Immigrant – Immigrant from different country of birth; Im-Intra-Im: Immigrant – Immigrant from the same country of birth

+ p-value <0.05; ++ p-value < 0.01; +++ p-value <0.001
